# Preclinical orofacial pain assays and measures and chronic primary orofacial pain research: where we are and where we need to go

**DOI:** 10.3389/fpain.2023.1150749

**Published:** 2023-05-24

**Authors:** Shirin Sadighparvar, Faez Saleh Al-Hamed, Reza Sharif-Naeini, Carolina Beraldo Meloto

**Affiliations:** ^1^ Integrated Program in Neuroscience, McGill University, Montreal, QC, Canada; ^2^ The Alan Edwards Centre for Research on Pain, McGill University, Montreal, QC, Canada; ^3^ College of Dental Medicine, QU Health, Qatar University, Doha, Qatar; ^4^ Department of Physiology and Cell Information Systems, McGill University, Montreal, QC, Canada; ^5^ Faculty of Dental Medicine and Oral Health Sciences, McGill University, Montreal, QC, Canada

**Keywords:** orofacial pain, primary pain, preclinical studies & animal models, temporomandibular disorder, trigeminal neuralgia, burning mouth syndrome (BMS)

## Abstract

Chronic primary orofacial pain (OFP) conditions such as painful temporomandibular disorders (pTMDs; i.e., myofascial pain and arthralgia), idiopathic trigeminal neuralgia (TN), and burning mouth syndrome (BMS) are seemingly idiopathic, but evidence support complex and multifactorial etiology and pathophysiology. Important fragments of this complex array of factors have been identified over the years largely with the help of preclinical studies. However, findings have yet to translate into better pain care for chronic OFP patients. The need to develop preclinical assays that better simulate the etiology, pathophysiology, and clinical symptoms of OFP patients and to assess OFP measures consistent with their clinical symptoms is a challenge that needs to be overcome to support this translation process. In this review, we describe rodent assays and OFP pain measures that can be used in support of chronic primary OFP research, in specific pTMDs, TN, and BMS. We discuss their suitability and limitations considering the current knowledge of the etiology and pathophysiology of these conditions and suggest possible future directions. Our goal is to foster the development of innovative animal models with greater translatability and potential to lead to better care for patients living with chronic primary OFP.

## 1. Introduction

Orofacial pain (OFP) is that localized below the orbito-meatal line, above the neck and anterior to the ears, including pain in structures of the oral cavity. According to the latest OFP classification, OFP can be acute, when it lasts for less than 3 months, episodic, when it occurs on fewer than 15 days per month whether or not for more than 3 months, or chronic, when it persists for more than 3 months and is present on at least 15 days per month (1). This distinction is important because chronic OFP is often accompanied by reduced quality of life, sleep and psychological disturbances and disability (2–6) that require different management and it has less favorable prognosis. The estimated prevalence of chronic OFP in the general population is of 10% (7–9). OFP can be additionally classified as primary, when its etiology is unknown (e.g., chronic primary temporomandibular joint pain and idiopathic trigeminal neuralgia), or secondary, when it has an identifiable cause (e.g., temporomandibular joint pain attributed to arthritis and trigeminal neuralgia attributed to multiple sclerosis). In other words, chronic primary OFP conditions can be understood as their own disease, while chronic secondary pain syndromes represent symptoms of other underlying conditions or diseases (10).

Some of the most challenging types of chronic primary OFP to manage include painful temporomandibular disorders (pTMDs), idiopathic trigeminal neuralgia (TN), and burning mouth syndrome (BMS). These seemingly idiopathic conditions have no cure and treatment is mostly palliative, geared toward pain management and coping strategies. Often, patients living chronic pain, including pTMDs, TN, and BMS, must endure significant biological, psychosocial, and economic burdens that also affect their families, and society as a whole (11).

The most common types of primary pTMDs are myofascial pain and temporomandibular joint pain (12). These conditions have a prevalence of about 5% that is greater in women than in men (13, 14) and their hallmark is spontaneous masticatory muscle and/or temporomandibular joint pain that is exacerbated upon jaw function. TN has a lifetime prevalence of 0.16%–0.3% and also affects more women than men (1.5:1) (15). TN is characterized by recurrent severe paroxysmal pain restricted to the territory of the trigeminal nerves (which innervate the orofacial region) lasting from a fraction of a second up to 2 min. TN pain is described as electric shock-like, stabbing, or sharp, and is triggered by innocuous stimuli (e.g., washing the face, eating, and brushing the teeth). BMS has a prevalence of 2.5%–5% in the general population that rises to 14% among post-menopausal women. BMS is characterized by a spontaneous burning sensation that most commonly affects the tongue, lips, and hard and soft palates and may be accompanied by an alteration of taste, a stinging sensation, dryness, and atypical odontalgia (16).

Despite considerable scientific advances over the past decades (17, 18), the need for greater understanding of the pathophysiology underlying these primary OFP conditions persists because treatment remains suboptimal. In this scenario, pre-clinical research has been and will continue to be an important and irreplaceable means of facilitating scientific advancement. Furthermore, we are currently experiencing a boom of both pain neuroimaging and genetic studies that find crucial complementation in animal research (19, 20).

The goal of this review is to describe the currently available rodent assays (injury models) and OFP measures (behavior) that can be used in support of chronic primary OFP research, in specific pTMDs, TN, and BMS. We will discuss their applicability and challenges considering our current knowledge of the etiology and pathophysiology of these conditions and possible future directions. Previous reviews have focused on other conditions linked to OFP, including migraine/headaches (20, 21) and oral cancer (22).

## 2. Animal assays

### 2.1. Chronic primary pTMDs

Chronic primary pTMDs have multifactorial etiology and complex pathophysiology that involve the biological [e.g., genetics (23), central sensitization (24)], psychosocial [e.g., stress and somatic symptoms (25)] and environmental [e.g., trauma (26)] realms (27). Mimicking this complex array of factors in animal models to enable studying the mechanisms leading to and sustaining pTMDs is clearly challenging. Hence, pre-clinical pTMD research has been mainly based on assays that induce pain to the orofacial region using the chemical, surgical, and jaw trauma assays described next (Table 1). This review does not include assays that modify occlusion (i.e., the way upper and lower teeth fit together) as a means of inducing pain the orofacial region because there is sufficient and consistent scientific evidence of the absence of causal relationship between occlusal factors and pTMDs (28).

**Table 1:** Pre-clinical rodent assays and OFP measures in support of chronic primary pTMD research.

	Species	OFP measures	Reference examples
**Chemical assays**			
Formalin injection	Rats and mice	Nocifensive behavior	(29–38)
CFA	Rats and mice	Mechanical sensitivity (von Frey filaments and bite force) Thermal sensitivity Facial expression Survival behavior Operant feeding behavior test Operant gnawing test	(39–56)
Carrageenan	Rats	Nocifensive behavior Operant thermal pain test	(57–63)
Capsaicin	Rats	Operant mechanical pain threshold test Operant thermal pain test Operant thermal and mechanical pain test	(64–67)
Mustard oil	Rats	Nocifensive behavior Mechanical sensitivity	(65, 68, 69)
Glutamate	Rats	Mechanical sensitivity	(70)
NGF	Rats	Nocifensive behavior Mechanical sensitivity	(59, 71)
**Jaw trauma assays**			
Repetitive contractions with jaw forced lengthening	Rats and mice	Muscle thickness and maximum jaw-opening distance*	(72–74)
Sustained mouth opening		Mechanical sensitivity	(75)
Ligation of the tendon of the anterior superficial part of the masseter muscle	Rats and mice	Mechanical sensitivity	(76, 77)
**Combined assays**			
COMT inhibition + jaw forced lengthening	Rats	Operant gnawing test	(78)

* Indirect measures of OFP; COMT, catechol-O-methyltransferase.

#### 2.1.1. Chemical assays

Here we briefly describe and discuss the most frequently used assays involving the injection of inflammatory and pain-inducing agents into the TMJ or masseter muscle to induce TMD-like pain in rodents. These assays are typically complemented with experiments assessing the OFP measure(s) of choice, which often include nocifensive behaviors, mechanical and thermal pain sensitivity tests, but can also include bite force and orofacial operant tests. Often developed and used to study mechanisms underlying pTMDs (29, 57, 79–82), the main limitation of chemical assays is their inability to sufficiently simulate the etiological and/or pathophysiological mechanisms of pTMDs. Moreover, except for Complete Freund's Adjuvant (CFA), these chemical agents induce pain that last for short periods of time. Nonetheless, the use of these assays has allowed the accumulation of critical evidence on the mechanisms of trigeminal nerve pain transmission (17). This knowledge forms the basis required to advance the methods employed in developing more clinically similar assays.

##### 2.1.1.1. Formalin injection

Introduced in 1989 (83), this is a reliable assay for inflicting OFP in rodents (29, 30). In this model, formalin (recommended concentration up to 1.5%) (31) is injected subcutaneously into the TMJ or masseter region (29, 32, 33). The reliability of this assay lies on its consistent elicitation of two phases of behavioral responses considered as OFP surrogate measures (e.g., face rubbing): an early phase lasting from 0 to 5 min post-injection and a late phase lasting from 10 to 50 min post-injection [times may vary according to strain (34)]. The duration of each phase may vary with the animal strain. At each phase, the duration of face rubbing is annotated and compared to that of saline-injected animals: prolonged face rubbing has a positive correlation with formalin concentration. Pain in the early phase is attributed to direct stimulation of sensory afferent neurons, while pain in the last phase has been proposed to reflect the combined effects of afferent input, central sensitization and inflammatory response (35). While the mechanisms underlying pain in the early phase can arguably parallel acute OFP in humans, as the latter is often the result of jaw trauma (36) (as opposed to the injection of a chemical), the mechanisms in the late phase may partly parallel those involved in chronic primary pTMDs, as central sensitization and pathways of immune and inflammatory response seem to contribute to these conditions (37, 38).

##### 2.1.1.2. Complete freund's adjuvant (CFA)

CFA is a valid irritating agent composed of desiccated mycobacterium in paraffin oil and mannide monooleate that induces a potent inflammatory response in rodents following local injection (84) that is accompanied by long-lasting hyperalgesia (i.e., exacerbated pain response to a painful stimulus) and allodynia (i.e., pain response to a non-painful stimulus) (79, 85). CFA can be injected directly into the masseter muscle (39, 40) or into the TMJ (41) to cause OFP: in addition to leading to mechanical (42, 43) and thermal orofacial hypersensitivity (44), it also leads to reduced biting force (45) and signs of spontaneous OFP as assessed using the Grimace Scale (46).

The long-lasting effects (>30 days) (47) of the CFA assay comprise its main advantage, and it is considered one of the few assays that allow the investigation of the “chronic” phase of pain. Nonetheless, the underlying immune and inflammatory processes sustaining OFP post-CFA injection may or may not partly reflect those of chronic OFP in humans, as subcutaneous accumulation of dead bacteria is hardly an OFP trigger in patients.

To quantify the CFA-induced hyperalgesia and/or allodynia, this assay is paired with experiments assessing the OFP measure(s) of choice (48, 49), which often include mechanical and thermal pain sensitivity tests (50), but can also include bite force(52, 51), spontaneous pain assessments (46), and orofacial operant tests (52).

##### 2.1.1.3. Carrageenan injection

Carrageenan is a seaweed extract used to induce inflammation in rodents since 1962 (86). Its inflammatory and pain-inducing effects may be strain-dependent (87), but generally lead to acute swelling and behavioral nociceptive responses (i.e., face grooming). The injection of carrageenan (usually 1%–1.5%) into the TMJ of rats has been used to investigate the inflammatory aspects of TMJ hyperalgesia, under the assumption that TMJ pain may result from an inflammatory episode (88). These studies have shown the involvement of P2X receptors (57, 58, 89), nerve growth factor (59), β-adrenoceptors (59–61), and the co-participation of peroxisome proliferator-activated receptors-γ (PPAR-γ) and *κ*/*δ* opioid receptors (62) in TMJ inflammatory pain.

Pain and inflammation due to the injection of carrageenan is short-lived, typically lasting no more than 14 days.

##### 2.1.1.4. Capsaicin injection

Capsaicin is the active ingredient in hot pepper, first isolated in 1846 (90). Trigeminal nerve afferents are predominantly peptidergic and enriched with transient receptor potential cation channel subfamily V member 1 (TRPV1) (91, 92). Capsaicin can sensitize and modulate TRPV1-positive trigeminal afferents and brainstem nociceptive neurons in the trigeminal subnucleus caudalis/upper cervical cord (Vc/UCC) (93–97). As early studies have suggested neurogenic inflammation as one of the putative mechanisms of injury leading to pTMDs (98, 99), the injection of capsaicin into the TMJ (80, 100) or masticatory muscles of animals (101) [and humans (102–106)] has been used to study neurogenic aspects of nociceptive pain that may be relevant to pTMDs. For instance, studies have shown that the injection of capsaicin into the TMJ of rats (80, 95, 108), as well as mustard oil (108) and glutamate (71, 95, 108), lead to peripheral sensitization that may contribute to the primary hyperalgesia or allodynic states characteristics of pTMD patients (109). This sensitization is attenuated by *N*-methyl-d-aspartate (NMDA) receptors antagonists and suggests the involvement of NMDA receptors in nociceptive trigeminal responses (80, 71, 108–111, 110). Sex differences in the orofacial nociceptive response of capsaicin-sensitive neurons also exist (111). Capsaicin cream has also been directly applied to the face of rats to induce mechanical pain sensitivity (64, 65).

##### 2.1.1.5. Mustard oil

The use of mustard oil to induce inflammation in the TMJ was first proposed based on the assumption that inflammation may be one of the possible contributing factors to pTMD (81), and on early studies showing that it activates small C-fibres (112) and induces inflammatory responses such as plasma extravasation (113) and neutrophil infiltration (114). The injection of mustard oil into the TMJ activates Vc/UCC neurons (115, 116) and elicits a short-lasting pain responses that are modulated by opioids and serotonin (117–121). Early studies using mustard oil injected into the TMJ have implicated mechanisms involving NMDA receptors (108, 119), other peripheral excitatory amino acid receptors (122), and neurokinins (123) in TMJ nociception, and peripheral and central γ-aminobutyric acid (GABA)-A receptors in TMJ anti-nociception (124, 125). These effects seem to vary between sexes (126–131) and throughout the estrous cycle (132).

Mustard oil has also been injected into the masseter muscle to induce short-lasting pain (68, 133), which likewise activates neurons in the trigeminal sensory nuclei (134) in a manner that involves peripheral NMDA receptors (135) and peripheral 2-amino-3-(3-hydroxy-5-methyl-isoxazol-4-yl) propanoic acid (AMPA) receptors (136). Studies using this model have also shown the involvement of TRPV1, the transient receptor potential ankyrin type 1 (TRPA1), and the transient receptor potential melastatin type 3 (TRPM3) in craniofacial muscle pain processing (65, 69).

##### 2.1.1.6. Glutamate injection

Glutamate is an endogenous neurotransmitter produced by both neuronal [including trigeminal ganglia neurons (137)] and non-neuronal cells (138–140) which acts via the centrally and peripherally expressed NMDA, AMPA, kainate, and metabotropic glutamate receptors. As such, glutamate has complex pain modulating roles centrally and pain inducing effects peripherally (141). Glutamate is long known for its involvement in OFP transmission (116, 122, 142) and transcriptome analysis has recently reaffirmed its role in inflammatory pain-signaling (143). Notably, glutamate seems to produce similar acute pain responses in humans and rats (144).

When injected into the masseter of rodents, glutamate-induced pain leads to both nociceptor activation and sensitization and local inflammation (70, 112, 144). Contrarily, glutamate injected into the TMJ [and other joints(145)] seems to have a limited inflammatory component (146).

##### 2.1.1.7. Nerve growth factor (NGF)

NGF is a neurotrophic protein that acts mainly via the tyrosine kinase receptor A (TrkA) and the p75 receptor (147). The suitability of this model for the study of TMD-related pain is largely based on experimental studies in healthy people showing that NGF injection into the masseter leads to long-lasting mechanical allodynia and hyperalgesia and to pain during strenuous jaw movements that are characteristic of pTMDs of muscular origin (148–150).

In rodents, intramuscular injection of NGF has been shown to sensitize masseter muscle afferent fibers without producing local inflammation (59, 151) in a manner that is at least partly mediated by enhanced peripheral NMDA receptor expression (71, 152) and increased glutamate expression in the sensory nerve endings in the muscle (82). Sex-related differences in nociception induced by peripheral NGF exist in both humans and animals (71, 148, 149).

#### 2.1.2. Jaw trauma assays

Jaw trauma, including sustained mouth opening and frequency of parafunctional behaviors, are known risk factors of pTMD onset (36, 153) and are associated with chronic pTMDs (26). Hence, assays based on jaw trauma represent more construct-valid (i.e., property of an assay that seems to have a biologic basis like that of patients) models of pTMDs. However, these assays are not as widely known nor as widely used as the chemical assays and future in-depth investigations of the pain mechanisms following these assays could shed further light into the mechanisms that sustain pain in humans.

##### 2.1.2.1. Repetitive contractions with jaw forced lengthening

Considering microtrauma, including repetitive parafunctional oral habits, as a contributor factor to pTMDs (referred to as myofascial pain syndrome of masticatory muscles at the time), the assay employing repetitive contractions with forced lengthening of the mice's masseter was first proposed in 1995 to induce pTMD in mice (72). The model was later adapted to rats (73, 74) and consists of restraining anesthetized animals in supine position with their jaw open and using an electrical stimulator to deliver a set of supramaximal stimuli to induce repetitive tetanic eccentric contractions to the masseter unilaterally. Stimuli are delivered every 30 s with 10 s of rest between stimuli for 20 min per session for 14 consecutive days. Control animals are anesthetized, restrained, and the electrode positioned into the masseter muscle (without delivering the electrical stimuli) for the same amount of time.

This assay leads to increases in muscle thickness and reduction of maximum jaw-opening distance, suggesting a phenotype consistent with masticatory myofascial pain (147). However, direct measures of OFP, such as sensitivity to mechanical stimuli or the display of nocifensive behaviors, have not been assessed in animals subjected to this assay and the assay's ability to induce long lasting pain also remains to be demonstrated.

##### 2.1.2.2. Sustained mouth opening

In this assay (75), a bite block is placed between the upper and lower front incisors of anesthetized mice to keep their mouth maximally opened for 1.5 h per day for 5 consecutive days. Starting on day 3, mice display mechanical allodynia in the masseter/TMJ region compared to controls (anesthetized animals that did not undergo sustained mouth opening) that seems to be maintained for at least 27 days after the last sustained mouth opening session. The administration of ibuprofen (nonsteroidal anti-inflammatory), amitriptyline (tricyclic antidepressant), pregabalin (antiepileptic), or morphine (opioid) either partially or completely reversed mechanical allodynia, suggesting that the pain induced by this assay involves a complex array of biological pathways, as is believed to be the case in chronic OFP patients. Notably, mice exposed to the sustained mouth opening assay also displayed a reduced intake of hard-pelleted chow accompanied by weight loss that was reverted after replacement with soft-pelleted chow. These may be considered as surrogate OFP measures that are paralleled in humans, as masticatory dysfunction is a hallmark of pTMDs. Hence, the sustained mouth opening assay may be a suitable alternative to investigate the “chronic” phase of OFP and has greater translatability.

#### 2.1.3. Ligation of the tendon of the anterior superficial part of the masseter muscle

Considering the short-lasting pain induced by other assays used in the study of pTMDs of muscular origin—from hours to few weeks at most—this assay has been developed to induce long-lasting pain in rats (76) and was later adapted to mice (77). It consists of anesthetizing and restraining animals in supine position with their mouth open and accessing, freeing, and tying their tendon of the anterior superficial part of the masseter muscle (TASM) with two chromic gut ligatures, 2-mm apart. Control animals receive the same procedure except for the TASM ligation. In rats, TASM ligation led to short-lasting local inflammation (2 weeks) that subsided after 8 weeks, but orofacial mechanical allodynia and hypersensitivity were maintained throughout 8 weeks. Furthermore, activation and upregulation of NMDA receptors in laminae I/II of Vc/UCC neurons consistent with somatotopy of the mandibular branch (V3) of the trigeminal nerve and hyperreactivity of Vc/UCC astrocytes and microglia were greater in TASM and sham-operated than in naïve animals and lasted longer in TASM than in sham-operated animals. Morphine and duloxetine (serotonin-norepinephrine reuptake inhibitor) administered at 8 weeks d displayed anti-hyperalgesic effects. Further studies employing this assay (but with timelines shorter than 8 weeks) have indicated that GABA-A receptors in trigeminal ganglia cells and Vc/UCC afferent terminals projecting to the orofacial region have OFP-inhibitory roles (154, 155) and that the use of bone marrow stromal cells (BMSC) (156–158) or of an engineered herpes virus (159) may be new alternatives for OFP control.

#### 2.1.4. Combined assays

In an effort to create an assay that induces pTMD in manner that better emulates the multifactorial etiology of these conditions in humans, a study combining a pTMD predisposing factor—inflammation induced by the injection of carrageenan in the TMJ or sustained inhibition of the catechol-*O*-methyltransferase (COMT) enzyme, classically known for its role in chronic pain (160–162), including OFP (163, 164)—with a precipitating factor—jaw forced lengthening—found that the combination of COMT inhibition with jaw forced lengthening was able to extend the time rats needed to chew through obstacles—a proxy measure of OFP sensitivity—compared to exposure to each factor alone after 2 weeks (78). The assay's ability to produce longer lasting OFP remains to be demonstrated. Nevertheless, the concept underlying the development of this assay is innovative and can lead to the development of construct valid animal assays of pTMDs.

### 2.2. Trigeminal neuralgia

TN is a very painful condition with severe, short-lasting, spontaneous and innocuous stimulus-evoked stabbing pain attacks in the face for which the development of more efficacious treatment options is highly warranted. Recently proposed taxonomies classify primary TN into classical or idiopathic TN (165, 166). Classical TN is characterized by the demonstration (in MRI or surgery) of vascular compression with morphological changes of the trigeminal nerve root entry zone (167–169). Its etiology seems to be linked to this neurovascular compression in some but not all cases (170, 171), as not all patients who undergo surgical decompression experience improvement (172). Proposed etiologies of idiopathic TN include genetic mutations in neuronal voltage-gated ion channel gain-of-function (173, 174), neural inflammation (175), and non-specific lesions in the brainstem (176–178). Centrally mediated pain facilitation and reduced descending pain inhibition are also non-confirmed contributors to TN pain (179–181). Existing assays in support of TN research are those that induce neurogenic pain via chemical injection or nerve injury (Table 2). The latter group, in particular assays that compress the trigeminal nerve root entry, seem to better approximate the pathophysiology of classical TN attributed to neurovascular compression.

**Table 2:** Pre-clinical rodent assays and OFP measures in support of chronic primary TN research.

	Species	OFP measures	Reference examples
**Chemical assays**			
Injection of cobra venom into the ION	Rats	Nocifensive behavior Mechanical sensitivity	(182, 183)
**Nerve injury assays**			
Chronic constriction injury to the ION	Rats and mice	Nocifensive behavior Survival behavior Mechanical sensitivity Thermal sensitivity Operant thermal pain test	(184–192)
Trigeminal inflammatory compression of the ION	Rats and mice	Mechanical sensitivity Thermal sensitivity	(193–200)
Chronic compression of the trigeminal nerve root	Rats	Survival behavior Mechanical sensitivity Thermal sensitivity	(201, 202)
Chronic compression of the trigeminal ganglion	Rats	Mechanical sensitivity	(203)
Demyelination of the trigeminal nerve root	Rats	Mechanical sensitivity	(204)

ION, infraorbital nerve.

#### 2.2.1. Chemical assays

##### 2.2.1.1. Injection of cobra venom into infraorbital nerve

This assay consists of exposing the infraorbital nerve (ION) at its rostral extent to inject cobra venom (or saline, for control) into the ION's nerve sheath (205). As consequence, animals display long-lasting (60 days) mechanical allodynia, spontaneous pain behaviors (e.g., increased face grooming and headshaking), fewer exploratory activities that is indicative of anxiety, and impaired spatial learning and memory function (182). Injection of cobra venom also leads to demyelination changes of the ION and medulla oblongata and was thus claimed to induce changes similar to those seen in classical TN (183). Treatment with pregabalin, a gabapentinoid believed to block calcium channels thereby reducing excitatory neuronal activity, improves different pain behaviors and suggests the neuropathic nature of pain resulting from the injection of cobra venom (183).

Although this assay clearly seems to induce neuropathic pain, whether the neurobiological mechanisms underlying pain are translatable to those of TN patients and relevant for the development of new therapies for patients is debatable.

#### 2.2.2. Nerve injury assays

Neuropathic pain, such as that seen in TN patients, is defined by pain caused by a lesion or disease of the somatosensory nervous system (206). With a seminal paper published in 1980 (207), nerve injury assays in support of TN research constrict (ligate) or compress the ION or trigeminal nerve to induce pain. Constriction or compression of the ION consistently leads to chronic pain and mood alterations that are consistent with the clinical presentation of chronic pain patients. However, there are important limitations to methods that employ chromic suture to ligate or compress nerves, as the resulting pain may be at least partially due to chromic-induced changes in the chemical milieu around the nerve (208).

While assays of nerve transection to branches of the trigeminal nerve also exist (209, 210), they are likely more suitable to the study of mechanisms underlying pain in post-traumatic neuropathies due to, for instance, motor vehicle accident, sporting injury, or dental procedures. Transection or lesions to the TN are not putative contributors to primary TN and these assays will not be describe here.

##### 2.2.2.1. Chronic constriction injury (CCI) to the ION

The CCI-ION (184) is the most widely used assay to induce neuropathic pain in the orofacial region with 167 citations on PubMed and has been adapted from the assay that induces a CCI to the sciatic nerve of rats developed by Bennett and Xie in 1988 (211). In this assay, following a midline scalp incision, part of the skull and nasal bone are exposed allowing access to the edge of the orbit. Orbital contents are then deflected, and the ION—a purely sensory nerve—is dissected free at its most rostral extent just caudal to the IO foramen. Two chromic gut ligatures 2 mm apart that reduce the diameter but do not occlude circulation are then loosely tied around the ION. Animals that undergo ION-CCI display increased face-grooming and freezing-like behavior, concomitant with a decrease in exploratory behavior that peak after surgery but remain different from controls for more than 4 months. Mechanical allodynia displays delayed onset and persists for approximately 80 days. Notably, animals also display extraterritorial pain that extends to the contralateral side of injury, an indicative of the involvement of central sensitization mechanisms that is believed to play an important role in chronic pain states, including OFP (212–214).

Studies employing the ION-CCI model have contributed to our understanding of how the trigeminal nerve responds to injury and have highlighted its uniqueness. As an example, dorsal root ganglion sympathetic sprouting that is seen post sciatic nerve injury is absent in the trigeminal ganglion (215, 216). Furthermore, CCI to the sciatic or ION induce differential gene expression in the dorsal root or trigeminal ganglia, respectively (217). These findings show important differences in the mechanisms of spinal vs. trigeminal pain and underscore the need to develop animal assays that are specific to TN.

Modifications to the ION-CCI assay have been proposed mainly to simplify the complexity of the required surgical procedure (185, 186). Examples include the chronic constriction injury of the distal ION (dIoN-CCI) (187). This leads to ipsi- and contralateral mechanical allodynia that lasts at least 9 weeks and is accompanied by anxiety-like behaviors that last at least 6 weeks (188). Constrictive assays were also developed for mice but their ability to produce long-lasting hypersensitivity and/or pain-like behaviors (185, 186) remains to be demonstrated.

##### 2.2.2.2. Trigeminal inflammatory compression of the ION

Originally developed in rats, performing the ION-CCI assay in mice is challenging due to the small operating space and abundant blood supply in their facial area. The trigeminal inflammatory compression (TIC) of the ION assay was developed in response to this shortcoming (193, 194). In this model (193), a 15-mm antero-posterior incision is made at the facial midline and the infraorbital muscle is dissected from the bone to allow a gentle contraction of the orbit. This allows the visualization and dissection of the ION at its most rostral extent in the orbital cavity and the placement of a 2 mm chromic gut suture (6–0) between the ION and the maxillary bone. As consequence, mice display a slight peripheral nerve demyelination accompanied by mechanical allodynia that lasts for at least 21 weeks (195). Cold but not heat-induced allodynia lasts at least 8 weeks (196–199). In addition, ION-TIC mice also display anxiety-and depression-like behaviors, a consistent feature of chronic OFP patients (162). The same research group recently developed the FRICT-ION (Foramen Rotundum Inflammatory Constriction Trigeminal Infra-Orbital Nerve) assay, in which the ION is more easily accessed using an intra-oral approach (200). The main advantage of this assay is that it does not generate any outward physical signs, allowing for blinded pre-clinical studies. This assay leads to spontaneous pain behaviors and ipsi- and contralateral mechanical allodynia that last at least 100 days. Anxiety and depression-like behaviors also develop within 3–6 weeks.

##### 2.2.2.3. Chronic compression of the trigeminal (CCT) nerve root

This assay was developed in response to clinical evidence that neurovascular compression of the trigeminal nerve root plays a role in the pathogenesis of TN (218–220). First, a small curved and antero-posterior skin incision is made above the rat's eye, allowing the fascia and muscles to be laterally dissected free and the lateral retraction of the contents of the orbit. As result, the ION can be visualized deep within the orbit on the infraorbital groove of the maxillary bone. Based on the determination that the distance between the inferior orbital fissure to the proximate junction of the trigeminal nerve root with the pons is of approximately 1.5 cm in adult rats, a round plastic filament with 0.1 cm in diameter is placed above the ION superior surface and inserted for approximately 1.2 com into the intracalvarium through the inferior orbital fissure (not to reach/damage the pons). The filament is inserted into the canal between the cerebral dura mater and the pars petrosa (temporalis bone) to cross the Meckel's cave and reach the trigeminal nerve root. Animals subjected to CCT, but not sham-operated animals, develop mechanical allodynia that lasts at least for 4 weeks, and exhibit face-grooming behavior consistent with OFP for 3 weeks (201, 202). Both CCT and sham-operated animals develop heat-hypersensitivity over the course of 4 weeks (201). Activation and increase in the numbers of Schwann cells, astrocytes (i.e., A1-astrocytes) and microglia/macrophages, as well as an increase in the infiltration of macrophages and lymphocytes in the trigeminal root zone are also seen 4 weeks following CCT, suggesting the contributions of neuroimmune cells to the pathogenesis of TN attributed to neurovascular compression (203, 221).

A method using crystals of a superabsorbent polymer placed next to the rat's trigeminal nerve root has also been developed but its ability to induce pain hypersensitivity and pain-like behaviors remains to be demonstrated (222).

##### 2.2.2.4. Chronic compression of the trigeminal ganglion

In this assay (203), rats are mounted into a stereotaxic frame and a 21 gauge cannula is inserted into the trigeminal ganglion for the injection of 8 µl of a 4% agar solution. Control (sham-operated) animals receive all surgical procedures without agar injection. Animals with the compressed trigeminal ganglion exhibited mechanical allodynia and hypersensitivity that lasted up to 40 days and longer than those of naïve or sham-operated animals. However, there was no evidence of TN-like paroxysmal pain in animals with the compressed nerve, as there were no group differences in the number of facial grooming episodes exhibited. Further studies employing this assay to induce TN-like pain have suggested that NMDA receptors, particularly their NR-2 subunits, play an important role in the central processing of TN pain and are potential targets for the development of new therapies for TN (223).

##### 2.2.2.5. Demyelination of the trigeminal nerve root

This assay was developed based on evidence from classical studies that proposed a causal relationship between TN and focal demyelination due to compression of the trigeminal nerve root (224, 225). To induce focal demyelination, lysophosphatidic acid (LPA; 3 μl; 1 nmol) is injected into the trigeminal nerve root of anesthetized and restrained rats. Control animals receive all surgical procedures with the injection of vehicle instead of LPA. Injection of LPA produced severe demyelination of the axonal portion of the trigeminal nerve root for at least 2 weeks that was completely recovered by day 160. Despite the recovery of the myelin sheath, mechanical allodynia persisted for approximately 130 days and mechanical hypersensitivity (i.e., hyper-responsiveness to pin-prick stimulation) lasted for approximately 100 days. LPA injection produced no effect in thermal pain sensitivity. The display of nocifensive behaviors that would represent a proxy measure of TN-like paroxysmal pain has not been investigated (204).

### 2.3. Burning mouth syndrome (BMS)

BMS is a painful condition often described as a burning, scalding, or tingling feeling in the mouth that may occur every day or intermittently for months or longer. Dry mouth and an altered taste in the mouth typically accompany the pain. Like other chronic primary pain conditions, the etiology of BMS is believed to be multifactorial (226). Clinical research over the last two decades showing small-fiber atrophy in the tongue and increased expression of TRPV1 and purinergic receptors (P2X3) ion channels in the epithelium of the lingual mucosa of BMS patients suggest a neuropathic component in BMS (227–230). As for primary pTMDs and TN, the incomplete knowledge of the etiology of BMS makes developing a translatable preclinical model challenging and there are currently no definitive animal models of BMS. Nevertheless, assays that induce oral symptoms resembling those of BMS patients have been put forward and will be discussed here (Table 3), as they may be useful in the investigation of the pathogenesis of BMS. Methods involving nerve transection that is not a factor at play in BMS have also been developed (231). The utility of these assays for shedding light into the underlying mechanisms of BMS is debatable and they will not be described here.

**Table 3:** Pre-clinical rodent assays and OFP measures in support of chronic primary BMS research.

	Species	OFP measures	Reference examples
Artemin over expresser	Mice	Operant drinking test	(232)
Topical application of 2,4,6-trinitrobenzene sulfonic acid	Mice	Thermal sensitivity	(233)

#### 2.3.1. Artemin over expresser (ART-OE) mice

This BMS mouse model was inspired by findings showing the increased expression of artemin, a member from the glial cell line-derived neurotrophic factor (GDNF) family, in the inflamed skin of wildtype mice and that this overexpression leads to hypersensitivity to noxious thermal stimuli, to increases in the expression of the pronociceptive ion channels TRPV1 and TRPA1 in neurons of the dorsal root ganglia, and to an increased heat-induced firing of C-fibers (234). These findings led to the creation of ART-OE mice that overexpress artemin in the tongue epithelium with the goal of assessing whether it would induce oral sensitivity. Trigeminal afferents of adult ART-OE mice display changes in their anatomical, physiological, and transcriptional properties that include hypertrophy of myelinated and unmyelinated fibers of the lingual nerve, and marked increases in the expression of TRPV1, TRPA1, and GDNF-family receptor alpha 3 (GFRα3; a receptor component for artemin) (232). These mice also reduced their consumption of water containing either capsaicin (a TRPV1 ligand) or mustard oil (a TRPA1 ligand) compared to wildtypes, a behavior consistent with increased oral sensitivity.

In accordance with the clinical findings of small-fiber atrophy in the tongue (227) and increased expression of TRPV1 in the epithelium of the lingual mucosa of BMS patients (228), this suggests that GFRα3/TRPV1 and artemin-responsive fibers are possible contributors to BMS and that ART-OE mice may be a suitable model for future investigations of the mechanisms underlying BMS.

#### 2.3.2. Topical application of 2,4,6-trinitrobenzene sulfonic acid (TNBS)

This assay was created as consequence of earlier studies showing a role for artemin in pain (233, 235), including in tongue pain (232), and clinical findings of a near 3-fold increase in the expression of artemin in the tongue of BMS patients (233). To emulate this increased expression of artemin in the tongue of mice, TNBS that is known to increase artemin expression in oral mucosa tissues (236) is applied topically to the tongue of mice. In specific, a cotton saturated with a TNBS 10 mg/ml suspension (or vehicle, for controls) is placed on the dorsum of the tongue for 1 h. As result, mice develop heat hyperalgesia that lasts at least 11 days and is inhibited by treatment with a TRPV1 antagonist or with an anti-artemin antibody (233). A longer follow-up of heat sensitivity has not been done, but heat sensitivity thresholds seem to be approaching baseline levels starting on day 11 and the ability of this assay to induce long-lasting tongue pain remains to be demonstrated. Notably, TNBS induced tongue pain in the absence of inflammation or nerve damage, consistent with what is seen in BMS patients. Following the induction of artemin expression in the tongue with TNBS, the number of trigeminal ganglion neurons expressing GFRα3 and TRPV1 increased and current induced by capsaicin in trigeminal ganglion neurons enhanced. These phenotypes were reversed upon treatment with an anti-artemin antibody.

These finds reinforce a role for artemin in tongue pain and may suggest that topical application of TNBS a may be useful for the study of tongue pain hypersensitivity associated with BMS and may be particularly relevant for the understanding of pain flare ups of chronic patients.

## 3. Orofacial pain measures

Following orofacial pain induction by means of the selected assay, the resulting pain must be assessed to confirm the desired phenotype (i.e., ensure that the assay produced pain), and to allow the quantification of pain and comparisons pre- and post-treatment and/or between groups. Assessing pain in rodents is more challenging than in humans, given the impossibility of verbal expression. Because of that, pain is inferred based on measures of pain-associated behaviors that can be evoked (i.e., reflexive behaviors in response to mechanical and thermal stimulation), non-evoked (i.e., changes in nocifensive, survival, and elective behaviors), or mixed (e.g., conditioned place preference, conditioned place aversion, and operant behaviors). Evoked behaviors are those elicited when an exogenous stimulus is applied by an experimenter at the time of measurement. Non-evoked behaviors are those elicited without an exogenous stimulus being applied by an experimenter at the time of measurement and are often referred to as spontaneous behaviors. Measures of mixed behaviors were developed to overcome issues related to the reflexive and innate nature of commonly assessed evoked and non-evoked pain measures, respectively. These issues relate to the over-reliance of evoked and non-evoked measures on simple spinal reflexes (that can even be evoked in decerebrate animals) (237) that do not require cerebral processing of nociception (238–240). Conditioning and operant behaviors are indirect measures of pain that involve higher levels of brain processing and require animals to make specific choices based on learning processes (as do humans) (241). Here, we describe the pain-associated behaviors used to infer OFP in rodents.

### 3.1. Evoked measures

#### 3.1.1. Mechanical sensitivity

Originally developed for assessing tactile sensitivity in humans (242, 243), von Frey filaments are widely used for measuring mechanical sensitivity in the skin of rodents. Von Frey filaments consist of a series of fibers (typically nylon) of equal length and gradually increasing diameter that are individually and perpendicularly attached to a hand-held base. Von Frey tests rely on the fact that the force (in grams) required to lightly bend each filament increases as the filaments' diameter increase. In the ascending/descending technique (184) developed for the orofacial region, following habituation, rats are individually placed in a small transparent cage to allow video recording and presented with a series of von Frey stimuli exerting forces of 2 gm, 9 gm, 16 gm, 4 gm, and 1 gm. The animal's response to each stimulus is scored from 0 to 4: 0, when the animal has no response to the stimulus; (1) when it detects the filament but displays no aversive behavior; (2) when it withdraws the head as an indication of a mild aversiveness to the filament; (3) when it escapes or attacks the filament as an indication of strong aversiveness; or (4) when it displays an uninterrupted series of at least three facewash strokes directed to the stimulated facial area as an indication of prolonged aversiveness. A mean score can then be calculated by averaging the response scores of each of the five stimuli.

The ascending technique (53) was sequentially proposed to overcome the potential for bias due to the subjectivity in the scoring system of the ascending/descending technique (184). Here, following a habituation period during which rats are trained to rest their heads on the experimenter's hand, testing starts with applying a small-diameter filament (typically 0.192 g for rats and 0.045 g for mice) five times perpendicularly to the skin over the masseter/TMJ region with a few seconds of interval. A response to the filament is defined by a head withdrawal in at least three of the five stimulations. If no response, the next thicker filament is used until the response threshold can be detected. The response threshold is defined as the lowest force needed to produce at least three head withdrawal responses in five stimulations. Alternatively, the response frequencies to a range of von Frey stimuli can be computed [(number of responses/number of stimuli) × 100%] and a stimulus-response frequency curve plotted. The EF_50_, defined as the von Frey filament force (g) that produces a 50% response frequency, can then be calculated using regression analysis and used as a measure of mechanical sensitivity (244): a smaller EF_50_ indicates greater sensitivity. The ascending technique, as originally described or with modifications (76, 156, 190, 246, 247) that include the use of electronic filaments (94, 247–251), is likely the most widely used to measure mechanical orofacial hypersensitivity in rodents, including in the tongue (252).

To assess OFP in an objective manner that better represents the patients' cardinal symptoms, a few methods have been developed to measure mice bite force as a proxy measure of orofacial mechanical allodynia. This is supported by clinical studies showing that bite force is significantly reduced in chronic OFP patients (253, 254). In the first of such methods developed for rodents (52, 255), rats were maintained on a water-restriction schedule and trained to deliver gradually increasing bite forces with the aid of a computerized system that triggered the delivery of water upon biting. Only successful bites (strong enough to reach the gradually increasing thresholds) resulted in water delivery. Rats can reportedly be trained to achieve the final target of 1.3 kg within 3–4 weeks. Post-training reductions in bite force (e.g., following CFA injection in the masseter) can be prevented by anti-inflammatory agents (52) and are indicative of orofacial mechanical allodynia.

A method has also been developed to assess bite force in mice with the advantage that because they exhibit aggressive voluntary biting, a training phase is not needed. In this method, mice are individually placed in a cylindrical tube with an opening at one end for accommodation of their head and a custom-made bite transducer is slowly moved towards them, naturally eliciting a bite. The bite force transducer consists of two parallel and 4.5–5 mm apart beams which deformation (due to biting) results in a proportional change in resistance that can be measured (in N or g) and represents the bite force. Reductions in bite forces can be prevented by anti-inflammatory agents and are indicative of greater pain sensitivity (47, 51, 77, 256). Variations of this method also exist but follow the same principle: reduced bite force equals increased pain sensitivity (257).

#### 3.1.2. Thermal sensitivity

Heat hypersensitivity has been assessed mainly in studies of neuropathic orofacial pain or tongue pain. In one of the earliest studies (190), a device to loosely restrain rats while allowing access to the rat's snout was designed. The device allows animals to easily withdraw or flick their snouts. For the test, a radiant heat stimulus that raises skin temperature above 45°C within 8.5 s is placed 10 cm from the stimulation site (vibrassal pad). A cut-off time is pre-determined to prevent tissue damage. Head withdrawal or snout flicking is detected by a photocell that terminates the stimulus and stops the timer, which defines the head withdrawal latency. Latency is determined three times on each side with 2 min intervals between tests, with shorter latency being suggestive of heat hypersensitivity. Slight variations of this method also exist (201, 258–261).

Thermal hypersensitivity can also be measured by applying a small thermal probe to the animal's vibrassal pad (189, 262, 263). For this, animals habituated to being restrained by the experimenter and to being presented with the probe at room temperature are presented with the thermal probe at higher temperature. A cut-off time is pre-determined to prevent tissue damage. Repeated trials with interval in between them are typically done to determine the animal's average withdrawal latency (i.e., time until they withdraw or flick the snout). Similar methods have also been developed to assess thermal hypersensitivity in the tongue (233, 252).

### 3.2. Non-evoked measures

#### 3.2.1. Nocifensive behavior

Nocifensive behaviors are time-limited involuntary rodent responses to the administration of an algogen (e.g., formalin, capsaicin) to a body part, such as the TMJ, masseter muscle, or vibrassal pad (193, 264). Moving the mandible in a chewing-like motion, rubbing the orofacial region, or flinching the head are examples of commonly measured orofacial nocifensive behaviors (29, 41, 265). Typically, following a habituation period, animals are placed in a test box/cage where they can be watched/recorded while moving freely. Results are expressed as the total amount of time spent doing a specific behavior (31) or as the number of specific behaviors counted over a pre-determined period (33). The amount of time/number of behaviors displayed are positively correlated with pain, and treatment with analgesic drugs inhibits the behavior (31).

#### 3.2.2. Facial expressions

Like humans, animals also can demonstrate pain via facial expressions (266). Scoring facial features associated with pain in rodents (i.e., orbital tightening, changes in ear position, nose/cheek aspect, and whisker direction) is another measure of orofacial nocifensive behavior (267, 268). This is done using grimace scales that have been developed for both mice and rats (269, 270). For that, following acclimation, one animal at a time is placed in a partially transparent cage and filmed before and after the injection of an algogen. A set of baseline and post-injection photographs is then presented to independent scores who rate each photo: a score of “0” indicates high confidence of the scorer that no facial feature associated with pain was present; “1” indicates either high confidence of a moderate appearance of a facial feature associated with pain, or equivocation over its presence or absence; “2” indicates high confidence that a facial feature associated with pain was present. Baseline and post-injection scores are averaged, and results are typically expressed as the mean change in score, with greater changes being indicative of more pain. This process can now be automated to eliminate human error and provide an objective, reliable, and rapid way of quantifying spontaneous pain and pain relief in mice (271).

#### 3.2.3. Survival behavior

Feeding is an activity of primary importance to the animal's survival that involves actions of the TMJs, masticatory muscles, and tongue. Different aspects of feeding have been measured to infer OFP under the assumption that pain in the orofacial structures negatively affects feeding. Pain-associated feeding behaviors include food intake and meal pattern (an indirect feeding measure). Food intake is usually given by tracking the amount (in g) of food consumed over a period, corrected for spillage (54, 55, 272). Reduced food consumption is taken as a proxy measure of pain, as it suggests reduced engagement of orofacial structures. Meal pattern, which requires using a computerized strategy to track meal size, meal duration, and interval in-between meals, is a complex feeding measure which results seem to correlate to the intensity of the injury (54, 272).

Grooming is another activity of primary importance to the animal's survival and encompasses all forms of care and attention to the body surface (273). Facial grooming behaviors include facial rubbing, lower lip skin/cheek rubbing or facial scratching. Measuring facial grooming involves, following habituation of the animals to the test environment, placing them in a transparent or partially transparent cage where they can be recorded while moving freely. Results are expressed either as the total amount of time spent face-grooming (191, 201) or as the number of face-grooming episodes over a pre-determined period (187). The amount of time and number of face-grooming episodes are positively correlated with pain, and treatment with analgesic drugs inhibits the behavior (187, 274).

### 3.3. Mixed measures

#### 3.3.1. Operant behaviors

Developing operant tests is challenging due to the difficulty in generating behaviors that are indicative of OFP after cerebral processing. These tests offer the advantage of avoiding the stress that is likely invariably experimented by animals in experimenter-initiated tests, which can affect pain-related outcomes (265, 275). Here we describe the operant assays that have been developed specifically to assess OFP.

##### 3.3.1.1. Operant thermal pain test

To assess operant thermal pain behavior and characterize orofacial heat pain sensitivity (63, 66, 192, 276), unrestrained rats or mice are placed in a cage with acrylic walls containing an opening in one wall lined with grounded metal tubing that serves as thermode. A spouted watering bottle filled with diluted sweetened condensed milk (reward) is placed just outside the opening, allowing animals to access to the reward through the spout when simultaneously contacting the thermode with their face. Following sessions in which animals are trained to obtain the reward while contacting the thermode at room temperature, temperature is increased and the data acquisition system is activated. The system records simultaneous spout and thermode contact as a “licking” event, which can be interpreted as a successful attempt at the reward; thermode contact only as a “facial contact” event, which can be interpreted as a failed attempt at the reward; and the duration of each facial contact. The amount of reward consumed is also tracked by the experimenter. The licking/facial contact events and facial duration/facial contact event ratios, the cumulative facial contact duration and reward intake can then be calculated and provide indirect measures of orofacial thermal pain sensitivity.

Using cold thermode temperatures, this method can also be used to assess cold hypersensitivity (277). Importantly, these operant measures reflect the animal's choice to obtain the reward despite the painful thermal stimulation, which involves learning cerebral processes.

##### 3.3.1.2. Operant thermal and mechanical pain test

The dual operant thermal and mechanical pain behavior employs a modification to the system described in the above subsection (Section 3.3.1.1) by simply adding a mechanical component (67). In specific, nickel titanium wires (0.010 or 0.007 inches in diameter) are attached horizontally to the cage opening that allows access to the spouted watering bottle filled with diluted sweetened condensed milk (reward). Thus, access to the reward requires simultaneous contact with the wires. Similar to the operant thermal pain behavior, the system records “licking” events, “facial contact” events and the duration of each facial contact. The licking/facial contact events and facial duration/facial contact event ratios, the cumulative facial contact duration and reward intake can then be calculated and provide indirect measures of orofacial mechanical pain sensitivity. This dual system allows investigators to directly assess and compare mechanical vs. thermal pain using the same outcomes.

##### 3.3.1.3. Operant mechanical pain threshold test

The same group who developed the operant thermal pain test and the dual operant thermal and mechanical pain test, sequentially developed the operant mechanical pain threshold test (64). The new test consists of placing animals in a cage that contains at one end a round aperture with a 360° array of looped 0.010 in stainless steel wires with a 0.7 in opening at the center that partially blocks access to a spouted watering bottle filled with diluted sweetened condensed milk (reward). Initially, the bottle spout is aligned with the array of wires so that initial contact with the bottle does not require contact with the mechanical stimulus. Upon contact with the bottle, an electric circuit is established triggering a motor that slowly moves the bottle away from the cage, requiring the animal to tolerate greater mechanical force to continue to receive the reward. This process is repeated five times or until 10 min of testing time elapse. The average tolerance distance can then be calculated for each testing day for each animal, with greater distances being indicative of reduced mechanical pain sensitivity. The main advantage of this test to the previously developed the dual operant thermal and mechanical pain test is that it allows the quantification of each animal's withdrawal threshold.

##### 3.3.1.4. Operant feeding behavior test

In the operant feeding behavior test (56), rats are trained to press on a bar four times to obtain one 45 mg food pellet. At the time of testing, the times of each reward obtained during a one-hour session and the inter-feeding interval are recorded. Results are shown in a histogram of feeding behavior with inter-feeding intervals binned at 5 s intervals, normalized to the total number of pellets consumed. A right shift in the distribution indicates food was consumed with larger inter-feeding intervals and suggests increased orofacial pain.

##### 3.3.1.5. Operant gnawing (chewing) tests

A hallmark of OFP conditions is functional pain, such as pain during mastication (278–281). The dolognawmeter, a device that allows the quantification of mice gnawing time (52), was developed considering that other operant tests assess noxious cutaneous stimulation rather than masticatory dysfunction. The dolognawmeter is a device designed to fit into a standard mouse cage and essentially consists of a 24 mm. internal diameter tube through which are placed two dowels part by 20 mm. Once a mouse is loaded into the dolognawmeter, an endcap is placed, and the mouse finds itself confined anteriorly by the series of two dowels. The mouse instinctively gnaws through them to exit the tube. Upon severing the first dowel, pistons retract it laterally and automatically start a timer that is stopped once the second dowel is severed. The amount of time taken to gnaw through the second dowel is an index of OFP: greater gnawing-time reflects increased OFP pain and can be brought back to baseline levels upon administration of analgesic drugs. Of note, mice use their front incisors to gnaw through the dowels and their tongue to remove debris from the mouth. This is different from humans to the extent that humans employ their molars for chewing. The dolognawmeter has been used mainly to study OFP linked to oral cancer (282–284) and has thus far been under-used in the field of non-cancer OFP (285). A modified dolognawmeter to test rats (the ratgnawmeter) has also been developed (78).

##### 3.3.1.6. Operant drinking test

Oral sensitivity, including tongue sensitivity, may be assessed based on drinking behavior. This test, modified from the paired-preference drinking aversion paradigm (286, 287), consists in attaching one bottle with water plus vehicle and another with capsaicin (1 µM) or mustard oil (100 µM) to the cage of mice housed individually for three days. Following 24 h during which animals have access to the two bottles (and to food) *ad libitum*, the volume consumed is recorded. At the end of each day, the bottle positions are changed to ensure findings are not due to a place preference. The average volume consumed over three days of testing and the ratio of capsaicin or mustard oil intake vs. total liquid intake (percentage) is then calculated and used as measures of oral sensitivity (i.e., lower volume and/or ratio are indicative of increased oral sensitivity) (232).

## 4. Discussion

It is hard to envision the much-needed advance in our comprehension of the etiological and pathophysiological mechanisms of chronic primary pain conditions and the consequent development of new drug therapies and treatment strategies to prevent and treat them without the support of pre-clinical research. For the success of this endeavor, it is imperative that appropriate pain assays and measures are selected to adequately address a study's goals. Another important aspect that requires careful consideration is the selection of study subjects (i.e., animal species, strain, sex, age, etc.). Previous reviews have discussed factors to consider in the selection of appropriate study subjects (239). In this review, we have presented the currently available pain assays and measures in support of chronic primary OFP, in particular pTMDs, TN, and BMS. Understanding how the current knowledge on these conditions has been constructed is important to enable the development of methods that will allow us to optimize the translation of findings into better OFP care.

Chronic primary pain conditions have seemingly idiopathic etiology and complex pathophysiology and there is robust scientific evidence that both processes are multifactorial and varied at the individual level (11, 17). It is thus challenging to envisage that a single preclinical pain assay might be necessary or sufficient to engage, in animals, etiological and pathophysiological mechanisms similar to those leading to the development and maintenance of pain in humans. Single assays, in particular those relying on an inflammatory or neuropathic injury, may at best represent environmental factors that produce nociceptive afferent inputs into the central nervous system (CNS) needed to initiate the painful state. An overwhelming majority of preclinical OFP research is, however, based on single assays. While these studies have been and will continue to be important to reveal critical inflammatory, neural, and immune aspects of OFP transmission and processing, their findings likely represent only an isolated fragment of the mechanisms involved in chronic pain. Hence, one of the current challenges to be overcome by preclinical OFP research is the development of integrated assays with greater construct validity that could potentially trigger the engagement of pain mechanisms paralleled in humans. Accordingly, a study has recently shown that OFP develops and is maintained for at least 2 weeks as result of the exposure of animals to a combination of assays mimicking a predisposing (i.e., pharmacological inhibition of COMT) and a precipitating pTMD factor (i.e., jaw forced lengthening) (78). Similar examples for idiopathic TN or BMS assays are not currently available.

The measures employed to infer OFP in rodents is another component of preclinical research design that requires careful consideration to enhance the translatability of findings. It would be ideal that the phenotype (i.e., a subject's observable traits) created by virtue of the selected OFP assay emulates as best as possible the symptoms linked with the disease of interest. In addition to the cardinal symptom of spontaneous (and often oscillating) pain, pain that is modified by jaw function (e.g., chewing hard food, laughing, yawning, resting the jaw) is a hallmark of pTMDs (278–281). However, preclinical studies investigating aspects of pain in the TMJs and masticatory muscles focus mainly on nocifensive behaviors and mechanical pain sensitivity measures. While the former has the advantage of being spontaneous (i.e., non-evoked) behaviors, they are elicited in response to the injection of an algogen and are short-lasting in nature. Conceivably, nocifensive behaviors are practical measures of acute pain sensitivity but do not sensibly represent chronic OFP symptoms in humans. Mechanical pain sensitivity is mostly assessed using von Frey filaments and seem to have face validity as a measure of pain evoked by palpation. Assessing palpation pain in the TMJs and masticatory muscles is an integral part of a pTMD clinical examination for adequately rendering a diagnosis [e.g., a myalgia diagnosis requires the patient to report that the pain elicited by palpation resembles the pain they experience outside the clinical setting, a.k.a. familiar pain (12)] and assessing severity and prognosis (288). Nonetheless, evoked pain is not the chief complaint of pTMD patients and their reason for seeking care. Furthermore, it has been speculated that von Frey tests assess noxious cutaneous stimulation rather than mechanical sensitivity. In the inability to communicate pain, it seems like preclinical measures of masticatory function, such as bite force (52, 51, 77, 255–265), feeding (54, 55, 272), and the operant gnawing tests (52, 78), would be more sensible measures of OFP resembling that of pTMD patients. Assessing these measures, however, does not come without challenges and disadvantages, which include the necessity of (sometimes extensive) animal training and of specific devices and computational systems to enable testing.

There are also no construct-valid animal assays that lead to behaviors that sufficiently mimic TN (i.e., long-lasting display of spontaneous pain attacks). It is commendable that many studies employing assays that compress the trigeminal nerve in a manner that likely partly parallels the pathophysiology of classical TN (167–169, 218–220, 224, 225) assess and show OFP for long periods of time lasting at least one month (201–204). These are, however, assessments of mechanical and/or thermal hypersensitivity and there is no reported evidence that these assessments trigger pain attacks. Pain attacks in TN patients are often triggered by innocuous stimuli, such as touching the face, brushing the hair, or simply stepping out into colder temperatures. Hence, it would be expected from a face-valid animal model of TN that the mechanical or thermal stimuli such as those delivered, for example, in von Frey or in thermal pain tests, would trigger pain attacks. The assay that injects cobra venom into the ION to induce TN seems to have greater face-validity despite lacking construct-validity, as injected animals display greater face-grooming behavior that could be indicative of spontaneous pain attacks compared to controls for at least 30 days. Going forward, it is important that pre-clinical studies of TN assess behaviors that could demonstrate the paroxysmal nature of pain, which could include, for instance, freezing behaviors in addition to face-grooming. Freezing is an instinctive behavior in animals to avoid physical pain (289) and is commonly assessed in pre-clinical migraine/headache research to infer pain (290) that would likely be informative for TN research.

It is also important to reiterate a previously voiced concern regarding the lack of specificity of assays supposedly relevant for TN, as the same assays (especially those compressing the infra-orbital nerve) are also used to induce painful trigeminal neuropathy. These are two distinct conditions both in terms of clinical presentation and evidence-based treatment and it is unlikely that the same assay would validly recreate pathophysiological mechanisms simultaneously relevant for both conditions (291).

Burning pain that can feel like one has scalded their mouth (more often in the tongue but may also affect gums, lips, the inside of cheeks, palate, and/or widespread areas in the mouth) is the main complaint of patients with BMS (292). Although a neuropathic component has been suggested for BMS (227–230) and introduced into animal assays (232), this condition also is believed to be multifactorial (226). Creating a rodent assay that better approximates the pathophysiology of BMS and results in a phenotype consistent with its clinical manifestations—in terms of both chief complaint and chronicity—will likely require combined approaches. Another challenge in BMS research is the development of methods that assess burning pain in a translatable manner. Acidic foods and liquids, such as tomatoes, orange juice, carbonated beverages, and coffee, typically worsen the burning sensation. A possibility could be to invest in the development of measures that assess the intake of these commonly consumed products as opposed to water containing potent TRPV1 and TRPA1 and ligands (e.g., capsaicin and mustard oil, respectively). Notably, a recent study showing that nociceptive nerves are required for enforced hematopoietic stem cells mobilization has developed a method in which mice are fed with spicy (capsaicin-infused) chow without affecting their daily food intake or body weight compared to regular chow that could potentially be useful as an outcome measure in pre-clinical BMS research (293).

Of note, this review does not wish to make a statement that measures of mechanical and thermal pain sensitivity are not adequate or needed to demonstrate a painful phenotype. In fact, pTMD (109, 214, 294, 295), TN (182, 297, 298), and BMS (298–301) patients are more sensitive to experimental mechanical and thermal pain locally and often in other parts of the body. Hence, pairing face-valid measures of OFP sensitivity with complementary measures of pain sensitivity is not only needed for a comprehensive phenotypic characterization but is also likely informative of the disease pathophysiology.

### 4.1. Perspectives and New applications

The multifactorial etiology and complex pathophysiology of chronic primary OFP conditions is a challenge to the translatability of pre-clinical research. The development of models in which long-standing pain results from the exposure to biological, psychological, and environmental factors seems to be required if we are to better understand the mechanisms that sustain pain and to develop treatment strategies that target these mechanisms. A recent study reports that mice with genetic background susceptible to pain (i.e., transgenic mice expressing reduced COMT levels) that are not more sensitive to pain at baseline develop pain in multiple body sites following combined exposure to stress (i.e., swim stress) and to an OFP-relevant environmental factor (i.e., surgery to remove lower molars bilaterally) that lasts more than 3 months and is of greater magnitude in females (302). This multiple assay-based animal model of chronic primary pain seems to be a step in the right direction and could be used to inspire new pTMD, TN, and BMS pre-clinical models.

In the abovementioned multiple assay-based animal model of chronic primary pain, animals also displayed depressive-like behaviors for longer than 3 months. Measures of psychological well-being are additional complementary measures that should be assessed to fully characterize OFP-related rodent phenotypes, as well as to assess pain duration and response to treatment. It is well-documented that chronic OFP patients, including those living with pTMD (25, 303, 304), TN (305, 306), and BMS (307–310), display a complex array of symptoms that includes psychological comorbidities and poorer quality of life. Naturally occurring elective behaviors such as grooming, playing, socializing, and nest building are increasingly accepted as indicators of rodent well-being (311–314). Equivalent behaviors in humans include social interactions and participation in physical activities, which are negatively affected by chronic pain (315–317), including pTMDs, TN, and BMS (26, 306, 319). Hence, complementing the study design with measures of elective behaviors may be crucial to assess the entirety of an animal's orofacial pain experience.

A plethora of preclinical assays have been used and have been helpful to advance our understanding of the mechanisms of OFP. Combined with evidence originating from studies in humans, it is now time to put effort into developing construct-valid OFP assays and face-valid OFP measures that may help uncover pathophysiological aspects of chronic OFP and enable the development of drug therapies and treatment strategies that may survive the translational ladder. In this regard, this review hopes to foster the development of innovative animal models with greater translatability and potential to lead to better care for patients living with chronic primary OFP.
